# Resting-State Brain Activity Dysfunctions in Schizophrenia and Their Associations with Negative Symptom Domains: An fMRI Study

**DOI:** 10.3390/brainsci13010083

**Published:** 2023-01-01

**Authors:** Giulia Maria Giordano, Pasquale Pezzella, Luigi Giuliani, Leonardo Fazio, Armida Mucci, Andrea Perrottelli, Giuseppe Blasi, Mario Amore, Paola Rocca, Alessandro Rossi, Alessandro Bertolino, Silvana Galderisi, Italian Network for Research on Psychoses

**Affiliations:** 1 Department of Psychiatry, University of Campania “Luigi Vanvitelli”, 80138 Naples, Italy; 2 Department of Basic Medical Science, Neuroscience and Sense Organs, University of Bari ‘Aldo Moro’, 70124 Bari, Italy; 3 Department of Medicine and Surgery, LUM University, 70010 Casamassima, Italy; 4 Department of Neurosciences, Rehabilitation, Ophthalmology, Genetics and Maternal and Child Health, Section of Psychiatry, University of Genoa, 16132 Genoa, Italy; 5 Department of Neuroscience, Section of Psychiatry, University of Turin, 10126 Turin, Italy; 6 Department of Biotechnological and Applied Clinical Sciences, Section of Psychiatry, University of L’Aquila, 67100 L’Aquila, Italy

**Keywords:** schizophrenia, negative symptoms, motivational deficit, expressive deficit, neural correlates, resting-state fMRI, orbitofrontal cortex, ventral caudate

## Abstract

The aim of the present study was to examine the neurobiological correlates of the two negative symptom domains of schizophrenia, the Motivational Deficit domain (including avolition, anhedonia, and asociality) and the Expressive Deficit domain (including blunted affect and alogia), focusing on brain areas that are most commonly found to be associated with negative symptoms in previous literature. Resting-state (rs) fMRI data were analyzed in 62 subjects affected by schizophrenia (SZs) and 46 healthy controls (HCs). The SZs, compared to the HCs, showed higher rs brain activity in the right inferior parietal lobule and the right temporoparietal junction, and lower rs brain activity in the right dorsolateral prefrontal cortex, the bilateral anterior dorsal cingulate cortex, and the ventral and dorsal caudate. Furthermore, in the SZs, the rs brain activity in the left orbitofrontal cortex correlated with negative symptoms (r = −0.436, *p* = 0.006), in particular with the Motivational Deficit domain (r = −0.424, *p* = 0.002), even after controlling for confounding factors. The left ventral caudate correlated with negative symptoms (r = −0.407, *p* = 0.003), especially with the Expressive Deficit domain (r = −0.401, *p* = 0.003); however, these results seemed to be affected by confounding factors. In line with the literature, our results demonstrated that the two negative symptom domains might be underpinned by different neurobiological mechanisms.

## 1. Introduction

Negative symptoms represent a key feature of schizophrenia [1,2,3,4]. They have a pivotal role in determining poor functional outcome and poor quality of life in subjects affected by schizophrenia [5,6,7,8,9,10,11]. In addition, there is currently no effective pharmacological or psychosocial treatment for these symptoms, in particular when they are primary and enduring [2,3,4,12,13,14,15,16,17,18,19]. As a result, negative symptoms continue to be an unsatisfied need in the care of people with schizophrenia, and pose a significant burden for patients, family members, and healthcare systems [2,4,20,21,22].

Negative symptoms represent a very heterogenous and complex construct; therefore, their conceptualization has been long at the center of debate. The current conceptualization of these symptoms, according to the Treatment Research to Improve Cognition in Schizophrenia (MATRICS) initiative developed by the National Institute of Mental Health (NIMH), comprises five symptoms, namely avolition, anhedonia, asociality, blunted affect, and alogia [23].

The most validated structure supports a two-factor model of negative symptoms, including the Motivational Deficit domain (avolition, anhedonia, and asociality) and the Expressive Deficit domain (blunted affect and alogia) [2,4,23,24,25,26,27], which are linked to different alterations in brain activity and connectivity within several areas and circuits [24,28,29,30,31,32,33,34].

The most recent pathophysiological theory subtending negative symptoms shows an association of the Motivational Deficit domain with dysfunctions in several features of motivation, which are often impaired in subjects affected by schizophrenia [24,30,35,36,37,38,39,40,41,42,43,44]. As a matter of fact, patients seem to have impairment in reward-related learning, as well as in the adaptive integration of value information with action selection [24]. These alterations might result from a dysfunctional connectivity between several brain areas along the motivational pathways.

These pathways are represented by two circuits: the “motivational value system or reward circuit” and the “motivational salience circuit” [24]. The first pathway comprises the ventral tegmental area and the ventro-medial substantia nigra pars compacta. Both of these regions project to the nucleus accumbens shell, the dorsal striatum, the medial orbitofrontal cortex, and the ventro-medial prefrontal cortex. The second pathway comprises the ventral tegmental area, the dorso-lateral substantia nigra pars compacta, and the nucleus accumbens core. This latter region projects to the dorsal striatum, the dorso-lateral prefrontal cortex, the ventro-lateral prefrontal cortex, and the dorsal anterior cingulate cortex [24,37,39,40].

Several task-related functional magnetic resonance imaging (fMRI) studies looked into the activation of brain areas implied in reward anticipation [29,45,46,47,48,49,50,51,52,53,54,55,56]. Some studies found an association between ventral striatum hypoactivation with the Motivational Deficit domain [29,46,47,48,49], anhedonia [50,51,52,53] or avolition [45,50,51], while no association has been reported with the Expressive Deficit domain. In addition, blunted ventral striatum activity was also found to correlate with the overall severity of negative symptoms, but also with positive symptoms, even after controlling for the effect of medications, and with depressive symptoms [29,45,48,54,55,56,57]. Therefore, it is possible that the association reported between negative symptoms and ventral striatum hypoactivation could be linked to confounding factors (especially positive symptoms or extrapyramidal side effects), that might be included in negative symptom ratings or might lead to secondary negative symptoms.

Two studies showed a hypoactivation of the dorsal caudate but normal activation of the ventral striatum during reward processes [30,41]. This dysfunction correlated with avolition in both studies [30,41]. Additionally, other fMRI studies reported an association between the Motivational Deficit domain and hypoactivation in the inferior frontal gyrus [58], the ventromedial prefrontal cortex [50,59], the anterior cingulate cortex and insula [46], and the right dorsolateral prefrontal cortex [60] during reward anticipation, and an association between the severity of negative symptoms and disturbed brain functional connectivity within the motivational value system in patients performing a reward-related learning task [61]. However, the latter was also related to positive symptoms [62] and thought disorder [63].

Other studies used resting-state fMRI, a technique that may be able to overcome issues related to the study of task-related activation/functional connectivity that could result in specious findings due to the poor intellectual capacities or memory impairments, which are frequently present in subjects with schizophrenia. It had been found that the severity of negative symptoms correlated with dysfunctions of the resting-state functional connectivity in different pathways related to motivation, such as the right ventral putamen-medial orbitofrontal cortex pathway [64], the cingulo-opercular pathway (which includes the dorsal anterior cingulate cortex, the anterior insula, the anterior prefrontal cortex, the inferior parietal lobule, the basal ganglia, the thalamus, and the cerebellum) [65], and the left dorsal caudate-dorsolateral prefrontal cortex pathway [66]. The Motivational Deficit domain showed a negative association with altered connectivity between the ventral tegmental area and the right ventro-lateral prefrontal cortex, the bilateral insular cortex, and the right lateral occipital complex [40]; altered connectivity in the precuneus [67]; and altered connectivity within the medial prefrontal and temporal pathways [68].

Overall, the above-mentioned results converge on a key role of the fronto-striatal pathway in the pathophysiology of negative symptoms, in particular the Motivational Deficit domain [40,64,65,66,67,68].

On the other hand, the Expressive Deficit domain has been less investigated than the Motivational Deficit domain. It is probably related to deficits in cognitive (both neuro- and social cognition) capacities, which are frequently impaired in patients [2,24,28,38,69,70,71,72,73,74,75], and to neurological soft signs, indicating that this domain might be associated with alterations in neurodevelopmental processes [2,24,38,76,77].

In particular, one of the main theories of causation of the Expressive Deficit domain and its component symptoms posits that the bases of this negative symptom domain are deficits in emotional identification and discrimination and, more generally, in abnormalities in the perception of nonverbal social cues [24,25], with a consequent inability to infer meaning from social situations and behaviors and to respond appropriately. 

Few fMRI studies explored neural correlates of the Expressive Deficit domain [2,28,31,38,78,79,80,81,82,83,84,85,86] and showed conflicting results with regard to the location and the extent of these brain alterations. For instance, associations were reported between the Expressive Deficit domain or its component symptoms and a hypoactivation of the anterior cingulate cortex during a reward-cognition interaction task [78], a hypo- or hyperactivation of the amygdala during a facial expression identification or perception task [79,80,81,82], a hypoactivation of the prefrontal cortex, the caudate nucleus, and the anterior cingulate cortex, or a hyperactivation of the hippocampus, the cerebellum, the anterior temporal pole, and the midbrain during an emotional processing task [83]. Very few resting-state fMRI studies were performed to investigate the neurophysiological bases of the Expressive Deficit domain. In particular, abnormalities in fronto-polar cortex connectivity were found to be correlated with the Expressive Deficit domain [84], or with blunted affect [85]. At present, the brain areas most probably involved in the pathophysiology of the Expressive Deficit domain are the cortical motor areas, the ventrolateral prefrontal cortex, the rostral anterior cingulate cortex, the amygdala, and the basal ganglia [28]. 

Overall, the investigation of the neurobiological underpinnings of the negative symptom domains has produced an intricate picture, mainly indicating associations between the Motivational Deficit domain and dysfunctions of brain areas within the motivational circuits. However, it is important to note that drawing conclusions on the results presented in the literature is very difficult. In particular, the different conceptualization of negative symptoms across studies and the use of different measures to evaluate these symptoms, which are frequently not in accordance with their present conceptualization, represent a main weakness of the literature. 

Indeed, most of the above-mentioned studies that investigated neural dysfunctions related to negative symptoms [41,45,52,53,55,56,59,61,62,63,65,67,79,80,81,82,83] used first-generation rating scales, such as the Positive and Negative Syndrome Scale (PANSS) [87] and the Scale for the Assessment of Negative Symptoms (SANS) [88] that had several limitations [1,2,25]. For instance, the PANSS negative subscale takes into account aspects not related to negative symptoms, such as stereotyped thinking, which is related to the disorganization dimension, and difficulty in abstract thinking, which is associated with cognition. The SANS comprises the attention subscale in the evaluation of overall negative symptom severity; the SANS assessment of blunted affect includes inappropriate affect that is related to disorganization and decreased spontaneous movements that are considered as unspecific and more pertinent to depression, whereas its assessment of alogia includes the poverty of speech content that could be associated with formal thought disorder [25]. Therefore, findings emerging from studies that used first-generation scales might be influenced by other elements that are not considered as negative symptoms. Furthermore, both scales do not differentiate between anticipatory and consummatory anhedonia, and both focus on behavioral observation but not on internal experiences in the evaluation of the Motivation Deficit domain [25].

These issues have been addressed with the introduction of second-generation instruments, for instance, the Brief Negative Symptoms Scale (BNSS) [89], which takes into account the present conceptualization of negative symptoms and offers distinct ratings for internal experiences and observed behaviors. 

To date, only a few studies have attempted to investigate the relationships between brain activity during resting state and the two domains of negative symptoms using cutting-edge instruments [29,48,58,60,78]. Therefore, in light of the above observations, the current study had two primary goals: 1) to investigate the differences between healthy controls (HCs) and clinically stable individuals with schizophrenia (SZs) with respect to the resting-state activity of brain areas relevant to the neurobiological mechanisms of negative symptoms, and 2) to investigate the associations of resting-state activity with negative symptom domains, which were evaluated with a state-of-the-art assessment instrument (BNSS). 

## 2. Methods

### 2.1. Participants

Sixty-six SZs and forty-nine HCs were enrolled across five Italian university psychiatric clinics that joined the Italian Network for Research on Psychoses (NIRP) [7].

The inclusion criterion was a diagnosis of schizophrenia according to DSM-IV, confirmed by the Structured Clinical Interview for DSM IV—Patient version (SCID-I-P). 

The following were listed as the exclusion criteria: (a) a history of head injury resulting in loss of consciousness; (b) a history of moderate-to-severe intellectual disability or neurological diseases; (c) a history of alcohol and/or substance abuse in the previous six months; (d) current pregnancy or breastfeeding; (e) an inability to provide informed consent; and (f) treatment modifications and/or hospitalization due to symptom exacerbation in the previous three months. 

For the HCs, additional exclusion criteria were current use of drugs with effects on the central nervous system, a personal history of psychiatric disorders, and 1st-degree familiarity for psychotic disorders. Each HC was screened with the SCID-I-Non-Patient version (SCID-I-NP).

All subjects were requested to provide a written informed consent to take part in the study after getting a thorough description of the study’s procedures. These procedures adhered to the Helsinki Declaration of 1975, as updated in 2008, and to the ethical requirements of the relevant national and institutional committees on human experimentation. This study was approved by the Ethics Committee of the Università degli Studi della Campania “Luigi Vanvitelli”—Azienda Ospedaliera Universitaria ”Luigi Vanvitelli”, A.O.R.N. “Ospedali dei Colli” and was approved by the Ethics Committee of the involved collaborating institutions.

### 2.2. Psychopathological Assessment

In the present study, the PANSS was used to assess positive, negative, and disorganization dimensions. In particular, the positive dimension was calculated according to Wallwork and colleagues [90] by adding the scores of the items “delusions” (P1), “hallucinatory behavior” (P3), “grandiosity” (P5), and “unusual thought” (G9); the negative dimension was assessed by adding the scores of the items “blunted affect” (N1), “emotional withdrawal” (N2), “poor rapport” (N3), “passive/apathetic social withdrawal” (N4), and “lack of spontaneity and flow of conversation” (N6); and the disorganization dimension was assessed with the PANSS item “conceptual disorganization” (P2), in order to prevent overlap with cognitive impairment [9]. 

Negative symptoms were assessed using the Italian version of the Brief Negative Symptom Scale (BNSS) [89,91]. The BNSS is a scale developed according to the recent conceptualization of negative symptoms, in line with the NIMH-MATRICS Consensus Statement on Negative Symptoms [23]. This scale explores all the domains of the negative construct, including avolition, anhedonia, asociality, blunted affect, and alogia, plus an additional aspect, “distress”, which evaluates the lack of normal experience of distressing and unpleasant emotions [23]. The scale includes 13 items and 6 subscales (5 negative symptom subscales that include anhedonia, asociality, avolition, blunted affect, and alogia, and a control subscale that includes distress). The ratings for each item range from absent (0) to moderate (3) to extremely severe (6) symptom. In the present study, the “distress” subscale was subtracted from the overall score to calculate the negative symptom total score [91]. The Motivational Deficit domain was obtained by adding the scores of the subscales of anhedonia, asociality, and avolition, and the Expressive Deficit domain was obtained by adding the scores of the alogia and blunted affect subscales. 

We also used the Calgary Depression Scale for Schizophrenia (CDSS) to evaluate depression [92] and the St. Hans Rating Scale (SHRS) to assess extrapyramidal symptoms [93]. For all these evaluations, higher scores indicated more severe symptoms.

### 2.3. MRI Data Acquisition and Pre-Processing

MRI evaluations were performed at five different sites and with six different 3 Tesla scanners. For all participants, we collected one sMRI and a resting-state-based functional MRI (rs-fMRI). For the sMRI, the T1-weighted structural images used the SPGR or MPRAGE sequences. Gradient-echo echo-planar imaging sequence was used to acquire images during the rs-fMRI acquisition (300 s, 150 volumes). For the sMRI, data processing was performed using the Computational Anatomy Toolbox 12 (CAT12, Structural Brain Mapping group, Jena University Hospital, Jena, Germany—http://www.neuro.uni-jena.de/cat12/, accessed on 20 May 2022) included in SPM12 (Statistical Parametric Mapping, Institute of Neurology, London, UK—https://www.fil.ion.ucl.ac.uk/spm/software/spm12/, accessed on 20 May 2022). The T1-weighted images were normalized on a standard brain (MNI152) using a diffeomorphic registration algorithm (DARTEL) and segmented into different tissue classes (gray matter, white matter, and cerebrospinal fluid) based on probability maps. All images were then modulated through Jacobian determinants to preserve initial volumes and smoothed with a 3 mm isotropic Gaussian filter. For the purpose of analysis, we used segmented gray matter images from sMRI, which reflect the gray matter volume (GMV) information of the whole brain. The quality-based inclusion criteria were as follows: an absence in the raw images of technical artifacts, such as blurring, ringing, wrapping, and incomplete head coverage, and an absence in the segmented images of excessive noise, poor image contrast, and/or inadequate boundaries.

To compensate for differences between the scanners in the MRI acquisition window, individual gray matter images were combined using the ImCalc toolbox in SPM12 with a multiplicative function in order to obtain a binary mask of voxels acquired only in each individual scanner. This mask containing only voxels common to all acquisitions (approximately 359,000 isotropic 1 mm voxels) was applied to all individual images. The resulting gray matter volume (GMV) maps were included in the multimodal group analyses. Individual total intracranial volume (TIV) was also calculated and used as a disturbance covariate in subsequent analyses.

The rs-fMRI data were preprocessed with SPM12. For each participant, functional volumes were realigned to correct for head movement. Individual motion parameters were extracted and used to calculate Friston 24 motion parameters. The realigned images were rescaled, co-registered to T1-weighted structural images, spatially normalized to a standard space (MNI 152), and masked using the gray matter mask. Finally, noise covariates, including Friston 24 head motion parameters, white matter signals, and cerebrospinal signals, were regressed and the images were smoothed with an isotropic 6 mm FWHM kernel. Wavelet despiking was performed to remove motion-related distortions. The quality-based inclusion criteria were as follows: an absence of scan artifacts and low head motion (translation > 3 mm, rotation > 3°, change in Framewise Displacement between volumes—FD > 0.05). The individual mean value of FD was calculated and used as a disturbance covariate in subsequent analyses.

### 2.4. ROI Selection

For the extraction of signal time courses in each anatomical district, we used the Human Brainnetome Atlas (BNA) [94]. The BNA atlas divided the brain into 246 regions of interest (ROIs), with 123 for each hemisphere, comprising 210 cortical and 36 subcortical ROIs. For each subject, we extracted the time courses from each of the 246 ROIs using the Data Processing Assistant for Resting-State fMRI (DPARSF) (http://www.rfmri.org (accessed on 20 May 2022)). The extracted data were then normalized within each subject by T-score transformation in order to minimize the global signal differences between subjects.

Starting from the 246 ROIs that emerged from the analysis, we selected 17 ROIs for each brain hemisphere, using an average value of the resting-state BOLD signal of areas belonging to the same brain region. The ROIs were the dorsolateral prefrontal cortex (DLPFC), the ventrolateral prefrontal cortex (VLPFC), the orbitofrontal cortex (OFC), the precuneus (PCun), the inferior parietal lobule (IPL), the temporo-parietal junction (TPJ), the superior temporal gyrus (STG), the ventral anterior insula (vaIC), the dorsal anterior insula (daIC), the posterior insula (pIC), the lateral occipital cortex (LOC), the dorsal anterior cingulate cortex (dACC), the amygdala (Amy), the nucleus accumbens (NaC), the ventral caudate (vCa), the dorsal caudate (dCa), and the putamen (Pu). The coordinates and the dimension of the ROIs are summarized in Table S1.

### 2.5. Statistical Analyses

Between-group comparisons of socio-demographic and activity of the ROIs were performed with χ^2^ and one-way analysis of variance (ANOVA) tests, according to the type of variable.

In order to evaluate the correlations of the ROIs’ activity for each brain hemisphere with negative symptoms, we performed correlation analyses using Pearson’s R correlation coefficient. Correlation coefficients between 0.10 and 0.29 in absolute value were interpreted as indicative of a weak linear correlation, from 0.30 to 0.49 as a moderate correlation, and from 0.50 to 1 as a strong correlation [95].

The associations between these variables were also evaluated using partial correlation analysis, correcting for confounding factors (positive symptoms, disorganization, depression, and extrapyramidal symptoms) that might affect the relationship between the ROIs’ activity and negative symptoms.

The Statistical Package for the Social Sciences (IBM SPSS Statistics), Version 25, was used to conduct the statistical analyses.

## 3. Results

### 3.1. Sample Characteristics

Sociodemographic variables (i.e., age, gender, and years of education) were assessed in the SZs and HCs groups. Four SZs and three HCs were excluded from the analysis due to missing data. Therefore, the analyses were conducted in one hundred and eight participants (62 SZs, 46 HCs).

Table 1 shows the demographic and clinical characteristics of the study sample. The SZ and HC groups differed in age (*p* = 6.3 × 10^−5^) and years of education (*p* = 6.0 × 10^−8^).

### 3.2. Group Comparison on Resting-State Activity

**Figure 1:**
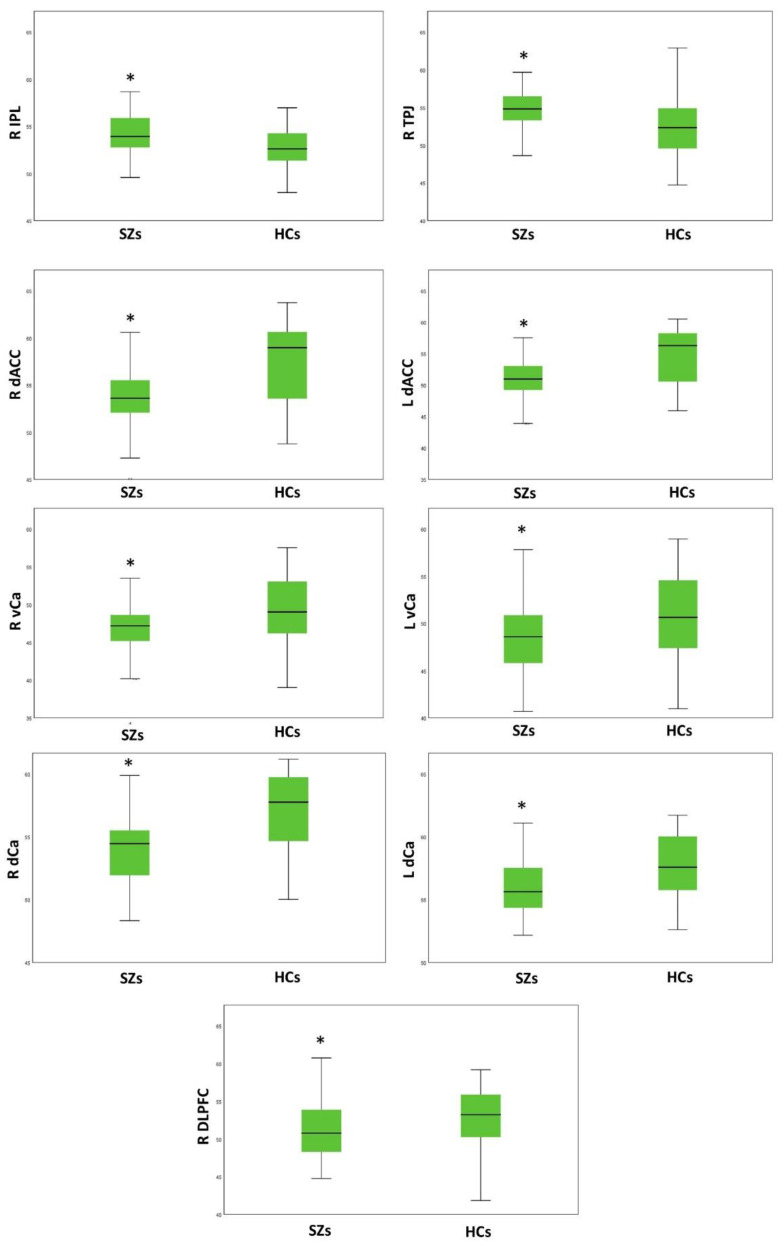
Group comparison on resting-state activity. R: right; L: left; IPL: inferior parietal lobule; TPJ: temporoparietal junction; daCC: dorsal anterior cingulate cortex; vCa: ventral caudate; dCa: dorsal caudate; DLPFC: dorsolateral prefrontal cortex. The SZs, compared to the HCs, exhibit a higher activity of the R-IPL and the R-TPJ, and a reduced activity of the bilateral dACC, the vCa, and the dCa, and the right DLPFC. * The asterisk flags the presence of a significant difference between the two groups in resting-state activity.

Between-group comparisons of the resting-state activity of the 17 ROIs for each hemisphere were performed using one-way ANOVA test, controlling for age. Statistical significance was set to *p* < 0.003 (*p* corrected for multiple tests). The SZ group, compared to the HCs, exhibited a higher activity of the R-IPL (*p* = 0.001) and the R-TPJ (*p* = 8.5 × 10^−5^), and a reduced activity of the right DLPFC (*p* = 0.002), the right (*p* = 6.24 × 10^−7^) and left (*p* = 5 × 10^−6^) dACC, the right (*p* = 3 × 10^−4^) and left (*p* = 0.003) vCa, and the right (*p* = 9.44 × 10^−8^) and left (*p* = 0.002) dCa (Table 2; Figure 1). In addition, the SZs and the HCs differed in the resting-state activity of other ROIs; however, these results did not survive correction for multiple tests (Table 2).

### 3.3. Correlation Analyses

Correlations between BNSS total score and resting-state ROI activity are shown in Table S2.

The correlations between the negative symptom domains and resting-state ROI activity are shown in Table 3. The Motivational Deficit domain showed a significant moderate correlation with the left OFC (r = −0.424, *p* = 0.002), while the correlation with the left IPL (r = 0.323, *p* = 0.020), left vCa (r = −0.367, *p* = 0.007), right (r = −0.343, *p* = 0.013) and left dCa (r = −0.346, *p* = 0.012) did not survive correction for multiple test (*p* > 0.003). The Expressive Deficit domain showed a moderate correlation with the left vCa (r = −0.401, *p* = 0.003), while the correlation with the right STG (r = 0.363, *p* = 0.008) and with the left OFC (r = −0.344, *p* = 0.013) did not survive correction for multiple tests (*p* > 0.003) (Table 3).

The five individual negative symptoms showed the same pattern of correlations with the negative symptom domains they belong (Table 4). In particular, the L-OFC correlated with asociality (r = −0.432, *p* = 0.001), avolition (r = −0.442, *p* = 0.001), and anhedonia (r = −0.333, *p* = 0.016), while the L-vCa correlated with blunted affect (r = −0.394, *p* = 0.004) and alogia (r = −0.378, *p* = 0.006).

### 3.4. Control Analyses

**Figure 2:**
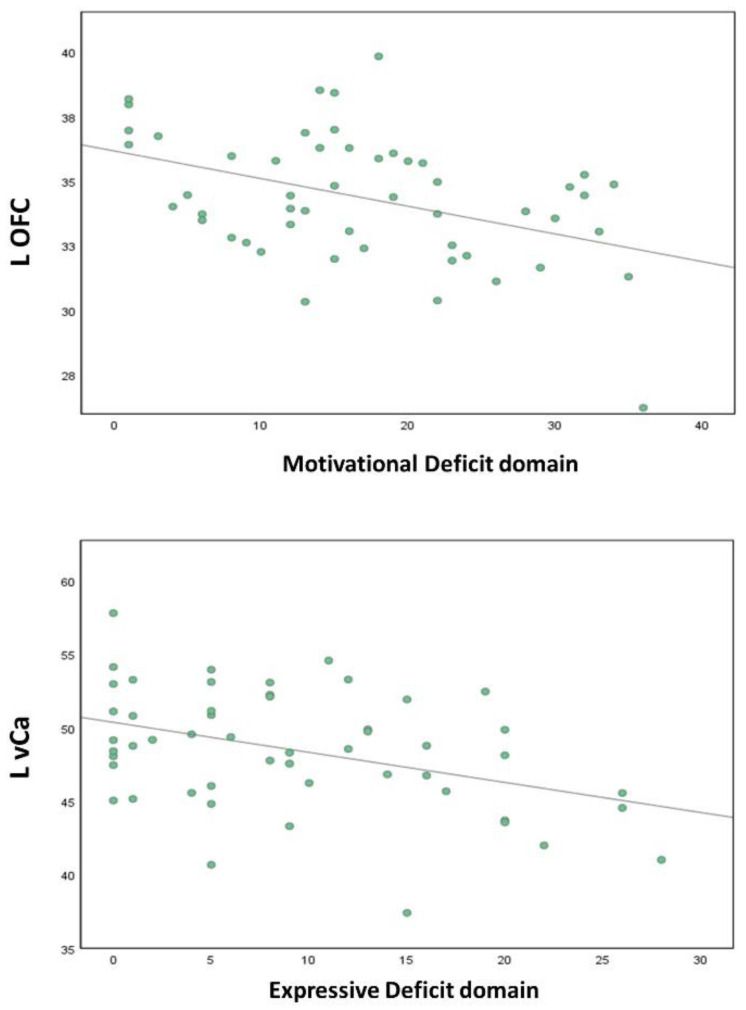
Correlations between the left orbitofrontal cortex and the left ventral caudate with the negative symptom domains. L: left; OFC: orbitofrontal cortex; vCa: ventral caudate. The left OFC correlates with the Motivational Deficit domain (correlations remain significant after controlling for the effects of the confounding factors), and the left vCa correlates with the Expressive Deficit domain (the *p*-values of these correlations are much higher than the original ones).

To rule out the possible effects of the confounding factors on our findings, we conducted control partial correlation analyses checking for the impact of the PANSS positive, the PANSS disorganization (PANSS item P2), the CDSS total score, and the SHRS Parkinsonism Global score (Table 4; Figure 2).

After controlling for these confounding factors, the correlations between the L-OFC and the BNSS total score (r = −0.436, *p* = 0.006), the Motivational Deficit Domain (r = −0.445, *p*= 0.005), avolition (r = −0.438, *p* = 0.007), anhedonia (r = − 0.372, *p* = 0.022) and asociality (r = −0.415, *p* = 0.011) still remained significant. The correlations between the L-vCa and the BNSS total score (r = −0.350, *p* = 0.031), the Expressive Deficit domain (r = −0.374, *p* = 0.021), blunted affect (r = −0.385, *p* = 0.017), and alogia (r = −0.333 *p* = 0.041) remained significant, although the *p*-values of these correlations were much higher than the original ones. The correlations between the left OFC and the Expressive Deficit domain and between the left vCa and the Motivational Deficit domain, which did not survive correction for multiple tests, were also affected by the confounding factors (Table 4). 

## 4. Discussion

The current study aimed to examine the neurobiological correlates of the two domains of negative symptoms, focusing on the brain areas that have been most commonly found in the literature as associated with these negative symptoms.

The two main goals were: (1) to determine the differences between the HCs and the SZs with respect to the rs functional activity in defined brain areas, and (2) to investigate the associations of resting-state activity with the two domains of negative symptoms, which were evaluated using the BNSS, an up-to-date assessment instrument in line with the current conceptualization.

The main results of our study included the following: (1) a higher activity of the right IPL and TPJ in the SZs, compared to the HCs; (2) a lower activity of the R-DLPFC, the bilateral dACC, the vCa, and the dCa in the SZs, compared to the HCs; (3) a relationship between the resting-state activity of the L-OFC with negative symptoms, in particular with the Motivational Deficit domain; (4) a relationship between the resting-state activity of the L-vCa with negative symptoms, in particular with the Expressive Deficit domain; (5) associations between the overall negative symptom severity with right STG, the right amygdala, the bilateral dCA, and the left vaIC, although these results did not survive correction for multiple tests.

Functional hyperactivation in the right IPL and TPJ has been previously reported in subjects with schizophrenia during the performance of a task and has been associated with the severity of psychotic symptoms [96,97,98,99]. Indeed, the TPJ and the IPL, especially in the right hemisphere, have a crucial role in understanding the source of sensory events [100,101,102]. In particular, these regions are involved in self/other distinction, which is the ability to distinguish between the representations of our own and others’ behaviors, experiences, and emotions [101,103,104,105]. However, these findings are not supported by other fMRI studies [106,107,108,109,110,111,112], of which the majority were task based [96,97,98,99,106,107,108,109,111,112], that reported a lower activation of these areas in SZs, compared to HCs.

In addition, our study reported a lower activity in the right DLPFC and the bilateral dACC in the SZs, compared to the HCs. Dysfunctions of both DLPFC [113,114,115,116,117] and dACC [118,119,120] have been frequently reported in schizophrenia. It has been hypothesized that the DLPFC has a critical role in executive, verbal working memory, and visual-spatial working memory [121]. Furthermore, this brain region is involved in the initiation and regulation of motivated behavior, and it integrates and transmits reward representations to the meso-cortico-limbic dopaminergic system, including the dACC [122,123]. The dACC is involved in cognitive control and integrates cognitive and emotional processes [124,125]; it also plays a critical role in updating prediction models, in both social and reward-related associative learning [126].

Furthermore, both DLPFC and dACC are involved in the motivational salience system, suggesting their role in the integration of motivational and cognitive information for goal-directed behavior [2,24,122], an aspect commonly altered in subjects with schizophrenia. However, we did not find a statistically significant direct or inverse correlation between these areas and the Motivational deficit domain or its component symptoms. Such areas might be associated with other domains of impairment, such as attention or working memory, but the investigation of these relationships is beyond the scope of our study.

Moreover, according to our findings, the bilateral vCa and the dCa have a lower activity in the SZs compared to the HCs. The caudate nucleus, a part of the striatum, is an integral component of the circuits involving the prefrontal cortex (VLPFC and DLPFC), and the OFC, playing an important role in cognition, movement, reward processes, and affect [127,128]. Our finding is in line with previous literature, since it has been reported that the caudate may be a contributor to the pathophysiology of the disease [14,31,129,130,131,132,133]. In addition, our finding could be read in light of the role played by the striatum (consisting of the vCa, the dCa, and the Pu) in cognitive processes through its interaction with the VLPFC, the DLPFC, and the posterior PFC. In particular, the dCa is connected to the DLPFC and is involved in monitoring and in the planning of an action; the vCa plays a role, together with the VLPFC, in the comparison between two or more actions (or stimuli) and in the selection of an action; the pathway between the putamen and the posterior PFC might be responsible of the execution of an action [134]. However, it would be of interest to note that a hyperactivity of the striatum has also been reported in subjects with schizophrenia [135,136,137]. This hyperactivation was related mainly to positive symptoms, and this might account for the differences from our finding [136].

With regard to the pathophysiological bases of negative symptoms, our study supported the hypothesis that the two negative symptom domains might show different neurophysiological correlates [24]. In particular, although the left OFC correlated with both negative symptom domains, the correlation between this region and the Expressive Deficit domain did not survive correction for multiple tests and was partially influenced by the confounding factors, while the correlation between this region and the Motivational Deficit domain survived correction for multiple tests and was not influenced by the confounding factors. Furthermore, the left vCa correlated with both negative symptom domains; however, the correlation between this region and the Motivational Deficit domain did not survive correction for multiple tests and the control for the confounding effects.

The relationship between Motivational Deficit and the OFC might be interpreted in light of the role of this brain region within the motivational circuit. Indeed, previous studies indicated that the OFC, especially in its medial part, is a key component of the motivational value system [24,37,39]. In particular, it has been demonstrated that the OFC is involved in reward processing, especially reward value encoding [138]. The OFC, together with the amygdala, (i) receives inputs from cortical areas that process the identity of stimuli, independently of their reward value, (ii) updates reward value representations and, then, (iii) projects to the anterior cingulate cortex to provide the reward outcomes for action–outcome learning [139,140,141], to the striatum for stimulus–response, habit and learning [142], and to the ventromedial prefrontal cortex to guide motivated goal-directed behavior [138]. It is responsible for generating and updating value representation, computing an outcome’s value, understanding if the outcome satisfies motivational needs, and comparing across alternative outcomes [143].

To our knowledge, this is the first rs-fMRI study that reported a negative association between the Motivational Deficit domain and the resting-state activity of the orbitofrontal cortex in schizophrenia. A previous rs-fMRI study found a negative correlation between this brain region and the global severity of negative symptoms [64], while an association with the Motivational Deficit domain was reported in structural MRI studies that found a correlation between this domain and decreased cortical thickness, decreased white matter integrity, and larger volume in the OFC [144,145,146]. The strength of our finding stems from fact that, as documented by the partial correlation analysis, this outcome was not mediated by positive symptoms, extrapyramidal side effects, disorganization, or depression, which frequently cause secondary negative symptoms. However, further investigations are needed since, in our study, the OFC correlated also with the Expressive Deficit domain, although, as already stated, this correlation did not survive correction for multiple tests and seemed to be influenced by the confounding factors (positive symptoms, disorganization, depressive symptoms, and parkinsonism), since the *p*-value of the partial correlation was higher than the original one.

On the other hand, we found that the severity of the Expressive Deficit domain and its component symptoms correlated with the resting-state hypoactivity of the L-vCa. This brain region is a key component of the ventral striatum and is involved in reward processing and affective functions [127], but also regulates executive functions by unifying cognitive processes, such as attention, planning, and decision making, through its connections with the DLPFC [147]. As mentioned in the Introduction, findings concerning neurobiological correlates of the Expressive Deficit domain are scarce and controversial [24]. Only one study, using task-related fMRI, reported a negative association between blunted affect, which belongs to the Expressive Deficit domain, and caudate nucleus hypoactivation [83].

However, it is important to note that the relationship between Expressive Deficit and the L-vCa that we found in our study seemed to be influenced by confounding factors, such as positive symptoms, disorganization, depressive symptoms, and parkinsonism, since the *p*-value of the partial correlation was much higher than the original one. This result is in line with the literature since previous studies reported an association between reduced ventral striatum activation and negative symptoms [29,46,47,48,49,50,51,52,53], both primary and secondary [57], and the severity of positive symptoms, even after controlling for the effect of medications, as well as depressive symptoms [29,45,48,54,55,56].

Our finding concerning the relationship between Expressive Deficit and the L-vCa might be also interpreted in light of the role played by this brain region in cognitive processes. Indeed, it is involved, through the DLPFC and the VLPFC, in working memory, in the comparison between two or more actions (or stimuli), and in the selection of actions [134,147,148]. Therefore, this result might support the hypothesis that the Expressive Deficit domain is subtended by deficits in cognitive functions [2,24,25].

Finally, even though the associations between the severity of negative symptoms and the right amygdala, bilateral dCA, and left vaIC activities did not survive correction for multiple tests, these results deserve an explanation. These brain areas are involved in motivated behavior and have already been reported in association with negative symptoms [24,28,31]. Indeed, the amygdala and the ventral anterior insular cortex play a critical role in modulating and mediating connections between the two motivational circuits and are involved in upgrading and retrieving value information to support motivated goal-directed behaviors [24,39]. The insular cortex, in turn, through the limbic regions such as the nucleus accumbens, transfers information to the dorsal striatum, which is connected to the cortical executive nodes, thus influencing goal-directed behaviors [35,149,150]. The dCa, a part of the dorsal striatum, is a constituent of the motivational value system. It is engaged in coding associations between actions/stimuli and outcomes in goal-directed behaviors and in selecting actions based on their currently predicted reward value [151]. In our study, we found an association between the dCa activity and the Motivational Deficit domain, but not the Expressive Deficit domain, although this result did not survive correction for multiple tests. This result is in line with previous literature that found a similar pattern of correlation [30,41,49]. However, it has been suggested that dCa activity is more prominent during the performance of a task [152,153,154]; therefore, it is possible that the lack of a strong association between negative symptoms and dCa in our study might depend on this aspect.

Our study has several strengths. Indeed, few studies have attempted to investigate the association between brain activity during resting state and negative symptoms using state-of-the-art instruments. We evaluated negative symptoms using the BNSS, a second-generation scale assessing negative symptoms according to their current conceptualization. Furthermore, we examined the neurobiological correlates of the two domains of negative symptoms, starting with the brain areas most commonly found in the literature to be associated with negative symptoms. In addition, our fMRI data were not recorded while the subjects performed a task, which decreased the possible confounding effects of cognitive impairments or poor intellectual abilities that often co-occur with negative symptoms.

Our findings should be also interpreted in light of some limitations. First, the sample size was relatively small, and a high number of correlations was performed, thus limiting the possibility of generalizing the results. Further studies with larger samples are needed to replicate these findings. In addition, although we performed control analyses checking also for parkinsonism that is an indirect measure of treatment, we could not check for the dose of antipsychotic medications that might influence the present results. Therefore, further studies including drug-naïve subjects are needed to confirm our findings.

In conclusion, the results of the present study, in line with the literature, support the hypothesis that the two negative symptom domains might show different neurophysiological correlates. Further studies aiming at investigating the pathophysiology of negative symptom domains, with the use more sophisticated techniques, such as machine learning analysis [155] and in the early stages of the illness, are strongly encouraged to promote knowledge in this field and foster the development of innovative treatment strategies.

## Figures and Tables

**Table 1:** Sociodemographic and clinical characteristics of the study sample.

	SZs (N = 62)	HCs (N = 46)	*p*
Age	37.92 ± 10.58	30.07 ± 8.11	**6.3 × 10^−5^**
Education	12.56 ± 3.15	16.37 ± 3.62	**6.0 × 10^−8^**
Gender (M/F)	37/25	25/21	0.580
PANSS Total score	60.20 ± 19.54		
PANSS Positive	7.59 ± 3.64		
PANSS Negative	12.87 ± 6.57		
PANSS Disorganization (item P2)	1.84 ± 0.97		
BNSS Total score	28.00 ± 17.61		
BNSS Motivational Deficit	16.98 ± 9.77		
BNSS Expressive Deficit	9.21 ± 7.94		
CDSS total score	3.95 ± 3.98		
SHRS global Parkinsonism	0.40 ± 0.88		
Type of AP medication (%)	77.4 % second-generation AP; 10.5% first-generation AP; and 12.1% both		

SZs: subjects with schizophrenia; HCs: healthy controls; PANSS: Positive and Negative Syndrome Scale; BNSS: The Brief Negative Symptom Scale; CDSS: The Calgary Depression Scale for Schizophrenia; SHRS: The St. Hans Rating Scale; AP: antipsychotic. *p* values in boldface indicate statistical significance.

**Table 2:** Group comparison on resting-state activity.

Brain Regions	SZs		HCs		F	*p*
	Mean	Standard Deviation	Mean	Standard Deviation		
**Right Hemisphere**						
DLPFC	51.55	4.04	52.94	3.93	10.029	**0.002**
VLPFC	51.61	2.34	50.73	2.47	3.786	0.054
OFC	35.18	3.19	37.05	3.61	5.847	0.017 *
STG	49.43	2.55	49.52	2.28	0.008	0.929
IPL	54.38	2.58	52.82	2.41	10.711	**0.001**
TPJ	55.18	2.88	52.49	3.76	16.723	**8.5 × 10^−5^**
Pcun	59.05	2.57	57.88	3.00	5.262	0.024 *
daIC	59.04	2.10	59.28	2.51	0.018	0.894
vaIC	57.00	3.52	56.23	3.82	1.093	0.298
pIC	58.76	2.40	59.04	2.41	0.179	0.673
daCC	54.27	3.85	57.74	3.92	28.201	**6.24 × 10^−7^**
LOC	50.28	3.75	47.97	4.34	7.842	0.006 *
Amy	50.71	5.39	48.62	5.50	3.653	0.059
NA	44.37	5.34	44.03	6.76	0.273	0.602
vCa	46.75	3.11	49.36	4.61	14.000	**3 × 10^−4^**
dCa	54.05	2.68	57.20	2.82	32.945	**9.44 × 10^−8^**
Pu	48.83	3.66	52.25	4.10	8.128	0.005 *
**Left Hemisphere**						
DLPFC	52.33	2.93	52.12	3.09	0.685	0.410
VLPFC	51.80	2.99	50.10	2.67	7.091	0.009 *
OFC	34.29	2.43	35.36	2.82	2.687	0.104
STG	52.88	3.13	52.87	2.29	0.171	0.680
IPL	52.38	3.18	52.34	2.58	0.275	0.601
TPJ	54.97	3.77	54.76	2.90	0.075	0.784
Pcun	57.50	1.92	57.51	1.82	0.215	0.644
daIC	59.20	3.07	59.58	2.15	0.005	0.941
vaIC	57.57	4.29	56.30	2.95	3.233	0.075
pIC	60.55	3.72	60.69	2.36	0.725	0.396
daCC	51.51	4.06	54.95	4.22	23.107	**5 × 10^−6^**
LOC	48.34	4.93	47.91	4.59	0.091	0.764
Amy	51.28	5.59	48.78	5.38	5.802	0.018 *
NA	45.55	3.52	46.60	3.96	1.011	0.317
vCa	48.39	3.81	50.62	4.64	9.465	**0.003**
dCa	55.88	2.46	57.70	2.57	9.950	**0.002**
Pu	50.10	3.87	53.24	3.42	5.617	0.020 *

SZs: subjects with schizophrenia; HCs: healthy controls; DLPFC: dorsolateral prefrontal cortex; VLPFC: ventrolateral prefrontal cortex; OFC: orbitofrontal cortex; STG: superior temporal gyrus; IPL: inferior parietal lobule; TPJ: temporoparietal junction; Pcun: Precuneus; daIC: dorsal anterior insular cortex; vaIC: ventral anterior insular cortex; pIC: posterior insular cortex; daCC: dorsal anterior cingulate cortex; LOC: lateral occipital cortex; Amy: amygdala; NA: nucleus accumbens; vCa: ventral caudate; dCa: dorsal caudate; Pu: putamen. In boldface, *p* =< 0.003 (*p*-value threshold corrected for multiple tests); * *p* =< 0.05.

**Table 3:** Correlations between BNSS domains and resting-state activity.

Brain Regions	BNSS Motivational Deficit		BNSS Expressive Deficit	
	Pearson’s Coefficient	*p*	Pearson’s Coefficient	*p*
R STG	0.178	0.208	**0.363**	0.008 *
R Amy	−0.260	0.063	−0.266	0.057
L OFC	**−0.424**	0.002 **	**−0.344**	0.013 *
L IPL	**0.323**	0.020 *	0.205	0.145
L vaIC	−0.284	0.041 *	−0.295	0.033 *
L vCa	**−0.367**	0.007 *	**−0.401**	0.003 **
R dCa	**−0.343**	0.013 *	−0.225	0.108
L dCa	**−0.346**	0.012 *	−0.240	0.086

BNSS: The Brief Negative Symptom Scale; OFC: orbitofrontal cortex; STG: superior temporal gyrus; IPL: inferior parietal lobule; vaIC: ventral anterior insular cortex; pIC: posterior insular cortex; daCC: dorsal anterior cingulate cortex; Amy: amygdala; vCa: ventral caudate; dCa: dorsal caudate; Pu: putamen. In boldface, correlations with r ≥ 0.300; * *p* =< 0.05; ** *p* =< 0.003 (*p* value threshold corrected for multiple tests).

**Table 4:** Correlations between negative symptoms with the left orbitofrontal cortex and the left ventral caudate.

	Left OFC		Left vCa	
	Pearson’s Coefficient	*p*	Pearson’s Coefficient	*p*
BNSS Total score	−0.420	0.002 *	−0.407	0.003 *
Motivational Deficit	−0.424	0.002 *	−0.367	0.007 ^#^
Avolition	−0.442	0.001 *	-	-
Asociality	−0.432	0.001 *	-	-
Anhedonia	−0.333	0.016 *	-	-
Expressive Deficit	−0.344	0.013 *	−0.401	0.003 *
Blunted affect	-	-	−0.394	0.004 *
Alogia	-	-	−0.378	0.006 *

BNSS: The Brief Negative Symptom Scale; OFC: orbitofrontal cortex; vCa: ventral caudate. Bold *p*-values are those statistically significant even after controlling for multiple test. * These correlations remain significant after controlling for the effects of the PANSS positive, the PANSS disorganization (PANSS item P2), the CDSS total score, and the SHRS Parkinsonism Global score (OFC: BNSS total score r = −0.436, *p* = 0.006; Motivational Deficit r = −0.445, *p*= 0.005; avolition r = −0.438, *p* = 0.007; asociality r = −0.415, *p* = 0.011; anhedonia r = −0.372, *p* = 0.022; and Expressive Deficit domain: r = −0.382, *p* = 0.018. vCa: BNSS total score r = −0.350, *p* = 0.031; Expressive deficit domain r = −0.374, *p* = 0.021; blunted affect r = −0.385, *p* = 0.017; and alogia r = −0.333 *p* = 0.041). ^#^ This correlation does not remain significant after controlling for the effects of the PANSS positive, the PANSS disorganization (PANSS item P2), the CDSS total score, and the SHRS Parkinsonism Global score (r = −0.309, *p* = 0.059).

## Data Availability

The original contributions presented in the study are included in the article/supplementary material, and further inquiries can be directed to the corresponding author/s.

## Supplementary Materials

The following supporting information can be downloaded at https://www.mdpi.com/article/10.3390/brainsci13010083/s1, Table S1: Regions of interest description; Table S2: Correlations between BNSS Total score and resting-state activity; Section: Members of the Italian Network for Research on Psychoses.
