# Supplementary material for: Resting-State Brain Activity Dysfunctions in Schizophrenia and Their Associations with Negative Symptom Domains: An fMRI Study
Source: Brain Sci. 2023 Jan 1;13(1):83. doi: 10.3390/brainsci13010083 (PMC9856573; doi:10.3390/brainsci13010083)
Supplement: Supplementary file 1 [file brainsci-13-00083-s001.zip › brainsci-2080332-supplementary.pdf]

Index

Table S1: Regions of Interest description

Table S2: Correlations between BNSS Total score and resting-state activity

Section: Members of the Italian Network for Research on Psychoses

Table S1. Regions of Interest description

Regions of Interest	Anatomical and modified Cyto-architectonic descriptions	lh.MNI(X,Y,Z)	rh.MNI(X,Y,Z)
**DLPFC, dorsolateral prefrontal cortex**	*A8dl, dorsolateral area 8*	[-18,24,53]	[22,26,51]
	A9l, lateral area 9	[-11,49,40]	[13,48,40]
	A9/46d, dorsal area 9/46	[-27,43,31]	[30,37,36]
	A46, area 46	[-28,56,12]	[28,55,17]
	A8vl, ventrolateral area 8	[-33,23,45]	[42,27,39]
**VLPFC, ventolateral prefrontal cortex**	*A44d, dorsal area 44*	[-46,13,24]	[45,16,25]
	IFS, inferior frontal sulcus	[-47,32,14]	[48,35,13]
	A45c, caudal area 45	[-53,23,11]	[54,24,12]
	A45r, rostral area 45	[-49,36,-3]	[51,36,-1]
	A44op, opercular area 44	[-39,23,4]	[42,22,3]
	A44v, ventral area 44	[-52,13,6]	[54,14,11]
**OFC, orbitofrontal cortex**	*A14m, medial area 14*	[-7,54,-7]	[6,47,-7]
	A12/47o, orbital area 12/47	[-36,33,-16]	[40,39,-14]
	A11l, lateral area 11	[-23,38,-18]	[23,36,-18]
	A11m, medial area 11	[-6,52,-19]	[6,57,-16]
	A13, area 13	[-10,18,-19]	[9,20,-19]
	A12/47l, lateral area 12/47	[-41,32,-9]	[42,31,-9]
**STG, superior temporal gyrus**	*A41/42, area 41/42*	[-54,-32,12]	[54,-24,11]
	TE1.0 and TE1.2	[-50,-11,1]	[51,-4,-1]
	A22c, caudal area 22	[-62,-33,7]	[66,-20,6]
	A22r, rostral area 22	[-55,-3,-10]	[56,-12,-5]
**IPL, inferior parietal lobule**	*A39c, caudal area 39(PGp)*	[-34,-80,29]	[45,-71,20]
	A40rd, rostrodorsal area 40(PFt)	[-51,-33,42]	[47,-35,45]
	A40c, caudal area 40(PFm)	[-56,-49,38]	[57,-44,38]
	A40rv, rostroventral area 40(PFop)	[-53,-31,23]	[55,-26,26]
**TPJ, temporo-parietal junction**	*A39rv, rostroventral area 39(PGa)*	[-47,-65,26]	[53,-54,25]
	A39rd, rostrodorsal area 39(Hip3)	[-38,-61,46]	[39,-65,44]
**Pcun, precuneus**	*A7m, medial area 7(PEp)*	[-5,-63,51]	[6,-65,51]
	A5m, medial area 5(PEm)	[-8,-47,57]	[7,-47,58]
	dmPOS, dorsomedial parietooccipital sulcus(PEr)	[-12,-67,25]	[16,-64,25]
	A31, area 31 (Lc1)	[-6,-55,34]	[6,-54,35]
**dACC, dorsal anterior cingulate cortex**	*A32p, pregenual area 32*	[-6,34,21]	[5,28,27]
	A24cd, caudodorsal area 24	[-5,7,37]	[4,6,38]
	A32sg, subgenual area 32	[-4,39,-2]	[5,41,6]
**vaIC, ventral anterior insular cortex**	*vIa, ventral agranular insula*	[-32,14,-13]	[33,14,-13]
**daIC, dorsal anterior insular cortex**	*dIa, dorsal agranular insula*	[-34,18,1]	[36,18,1]
	vId/vIg, ventral dysgranular and granular insula	[-38,-4,-9]	[39,-2,-9]
**pIC, posterior insular cortex**	*G, hypergranular insula*	[-36,-20,10]	[37,-18,8]
	vIa, ventral agranular insula	[-32,14,-13]	[33,14,-13]
	dIa, dorsal agranular insula	[-34,18,1]	[36,18,1]
**LOC, lateral occipital cortex**	*mOccG, middle occipital gyrus*	[-31,-89,11]	[34,-86,11]
	V5/MT+, area V5/MT+	[-46,-74,3]	[48,-70,-1]
	OPC, occipital polar cortex	[-18,-99,2]	[22,-97,4]
	iOccG, inferior occipital gyrus	[-30,-88,-12]	[32,-85,-12]
	msOccG, medial superior occipital gyrus	[-11,-88,31]	[16,-85,34]
	lsOccG, lateral superior occipital gyrus	[-22,-77,36]	[29,-75,36]
	mAmyg, medial amygdala	[-19,-2,-20]	[19,-2,-19]

**Amy, amygdala**	*lAmyg, lateral amygdala*	[-27,-4,-20]	[28,-3,-20]
**NaC, nucleus accumbens**	*NaC, nucleus accumbens*	[-17,3,-9]	[15,8,-9]
**vCa, ventral caudate**	*vCa, ventral caudate*	[-12,14,0]	[15,14,-2]
**dCa, dorsal caudate**	*vCa, dorsal caudate*	[-14,2,16]	[14,5,14]
**Pu, putamen**	*vmPu, ventromedial putamen*	[-23,7,-4]	[22,8,-1]
	dlPu, dorsolateral putamen	[-28,-5,2]	[29,-3,1]

Table S2. Correlations between BNSS Total score and resting-state activity

Brain regions	BNSS Total score	
	Right hemisphere	
	Pearson's coefficient	p
**DLPFC**	0.181	0.198
**VLPFC**	0.117	0.407
**OFC**	-0.162	0.25
**STG**	0.280	0.044*
**IPL**	-0.076	0.591
**TPJ**	-0.129	0.364
**Pcun**	-0.050	0.723
**daIC**	-0.094	0.506
**vaIC**	-0.271	0.052
**pIC**	-0.132	0.351
**daCC**	-0.019	0.893
**LOC**	0.024	0.864
**Amy**	-0.287	0.039*
**Nacc**	-0.183	0.194
**vCa**	-0.209	0.136
**dCa**	**-0.309**	**0.026***
**Pu**	-0.133	0.346
	Left hemisphere	
	Pearson's coefficient	p
**DLPFC**	0.186	0.187
**VLPFC**	-0.064	0.653
**OFC**	**-0.420**	**0.002****
**STG**	0.159	0.26
**IPL**	0.290	0.037*
**TPJ**	0.239	0.088
**Pcun**	0.047	0.742
**daIC**	-0.112	0.429
**vaIC**	**-0.309**	**0.026***
**pIC**	-0.090	0.525
**daCC**	-0.119	0.401
**LOC**	0.199	0.158
**Amy**	-0.225	0.109
**Nacc**	-0.047	0.739
**vCa**	**-0.407**	**0.003****
**dCa**	**-0.318**	**0.021***
**Pu**	-0.167	0.238

BNSS: The Brief Negative Symptom Scale; DLPFC: dorsolateral prefrontal cortex; VLPFC: ventrolateral prefrontal cortex; OFC: orbitofrontal cortex; STG: superior temporal gyrus; IPL: inferior parietal lobule; TPJ: temporoparietal junction; Pcun: precuneus; daIC: dorsal anterior insular cortex; vaIC: ventral anterior insular cortex; pIC: posterior insular cortex; daCC: dorsal anterior cingulate cortex; LOC: lateral occipital cortex; Amy: amygdala; NA: nucleus accumbens; vCa: ventral caudate; dCa: dorsal caudate; Pu: putamen.

In boldface correlations with r≥0.300; * p= < .05; ** p= < .003 (p value threshold corrected for multiple tests).

Section: Members of the Italian Network for Research on Psychoses

Members of the Italian Network for Research on Psychoses who participated in this study include: Paola Bucci, Giuseppe Piegari, Eleonora Merlotti, Daria Pietrafesa, Francesco Brando, Edoardo Caporusso, Noemi Sansone, Antonio Melillo (University of Campania "Luigi Vanvitelli", Naples); Marco Papalino, Vitalba Calia, Raffaella Romano (University of Bari); Pietro Calcagno, Martino Belvedere Murri, Simone Cattedra (University of Genoa); Cristiana Montemagni, Cecilia Riccardi, Elisa Del Favero (University of Turin); Francesca Pacitti, Rodolfo Rossi, Valentina Socci (University of L'Aquila).
